# Low-dimensional genotype-fitness mapping across divergent environments suggests a limiting functions model of fitness

**DOI:** 10.1101/2025.04.05.647371

**Published:** 2025-04-09

**Authors:** Olivia M. Ghosh, Grant Kinsler, Benjamin H. Good, Dmitri A. Petrov

## Abstract

A central goal in evolutionary biology is to be able to predict the effect of a genetic mutation on fitness. This is a major challenge because fitness depends both on phenotypic changes due to the mutation, and how these phenotypes map onto fitness in a particular environment. Genotype, phenotype, and environment spaces are all extremely complex, rendering bottom-up prediction unlikely. Here we show, using a large collection of adaptive yeast mutants, that fitness across a set of lab environments can be well-captured by top-down, low-dimensional linear models that generate abstract genotype-phenotype-fitness maps. We find that these maps are low-dimensional not only in the environment where the adaptive mutants evolved, but also in more divergent environments. We further find that the genotype-phenotype-fitness spaces implied by these maps overlap only partially across environments. We argue that these patterns are consistent with a “limiting functions” model of fitness, whereby only a small number of limiting functions can be modified to affect fitness in any given environment. The pleiotropic side-effects on non-limiting functions are effectively hidden from natural selection locally, but can be revealed globally. These results combine to emphasize the importance of environmental context in genotype-phenotype-fitness mapping, and have implications for the predictability and trajectory of evolution in complex environments.

## Full Text Availability

The license terms selected by the author(s) for this preprint version do not permit archiving in PMC. The full text is available from the preprint server.

