# Supplementary material for: Low-dimensional genotype-fitness mapping across divergent environments suggests a limiting functions model of fitness
Source: bioRxiv. 2025 May 31:2025.04.05.647371. Originally published 2025 Apr 9. Preprint. [Version 2] doi: 10.1101/2025.04.05.647371 (PMC12026818; doi:10.1101/2025.04.05.647371)
Supplement: Supplement 1 [file NIHPP2025.04.05.647371v2-supplement-1.pdf]

bioRxiv preprint doi: https://doi.org/10.1101/2025.04.05.647371; this version posted May 31, 2025. The copyright holder for this preprint (which was not certified by peer review) is the author/funder, who has granted bioRxiv a license to display the preprint in perpetuity. It is made available under aCC-BY 4.0 International license.

SUPPLEMENTAL INFORMATION (SI)

1. GENERATING δX CORRELATION MATRICES AND ASSESSING EXE INTERACTIONS

To further explore the gxExE interactions we discovered in Section I.B, we asked whether the δX of all mutants in two different environments were correlated with each other, and generated a correlation coefficient, or Pearson's r, with `scipy.stats.pearsonr`. Then, we can specifically zoom in on pairwise comparisons between the same perturbation on different bases, and ask whether these look more or less correlated than different perturbations on the same base. For example, in Fig. S2a, we show the effect of adding the 0.5% ethanol perturbation on top of each base, for all mutants. We find that in general, there is a positive correlation across the different bases. In contrast, when we added 4 uM H89, a drug, to our bases, we find that its effect is actually anti-correlated between the Salt base and the other two bases (Fig. S2b). To capture all of these effects, in Fig. S2c and d, we show the correlation matrices of environment to environment δX correlation for all pairs of environments. We first clustered rows of this matrix by perturbation type, and then by batch (panel c). Below, we present the same matrix with a different clustering. We first clustered mutants by base, and then by batch (d). We included these partitions in Fig. S2c and d as dashed lines on top of the correlation matrix. We find that the base-clustered matrix appears to have much more structure, and in particular block diagonal structure, than the perturbation-clustered matrix. This further supports the idea that the effect of environmental perturbations is heavily determined by the base.

To quantify the block-diagonality of each matrix, we used a tool from network theory called the "modularity" score, Q. Typically used to quantify the modularity of a network, Q can intuitively be understood as a measure of how much more density falls within a partition than would be expected of a random matrix, where the row sums are conserved. First, we scaled the values of the correlation matrices, which initially lay between -1 and 1, to fall between 0 and 1. Then, for each partition of the data, we calculated the difference between the observed and expected weight under a model of randomly distributed weights. As the matrix approaches perfect block-diagonal form, Q approaches 1. We calculated Q for the scaled correlation matrix with perturbation partitions and base-environment partitions, and found that as expected, Q is significantly higher for the base partition. We obtained values of Q=0.35 for the base-clustered matrix, and Q=0.08 for the perturbation clustered matrix.

2. SVD ON ADDITIONAL MUTANT SETS

We quantified the dimensionality of the rest of our "biological replicates" to include in Fig. 4c from scree plots like in Fig. 4a. We obtained estimates for detection limit as we described in the main text, using noise-only matrices. In Fig. S3, we present the scree plots that were the source of these estimates.

3. ALTERNATIVE WAYS TO QUANTIFY DIMENSIONALITY

We explored two other approaches to quantifying dimensionality. First, instead of allowing each base to have its own limit of detection, we set an overall limit of detection at the maximum fraction of variance explained by any of the noise matrices, yielding a more conservative estimate. In Fig. S4a, we show the inferred dimensionality using this "overall" detection limit. Our dynamic range is much smaller, but the conclusion from the main text does not change. We do not see lower dimensionality in the evolution base.

The number of dimensions that fall above a threshold is one way to quantify "dimensionality," but it is a discrete value. Another way to capture the scree plots in Fig. S3 is to calculate the entropy of the scree plot for the "real" components. To calculate Shannon entropy, we use the standard formula for the entropy of a distribution,

$$H = -\sum_i p_i \log p_i \tag{S1}$$

Here, we use the "distribution" of variance explained, so

$$p_i = \frac{\sigma_i^2}{\sum_j \sigma_j^2} \tag{S2}$$

bioRxiv preprint doi: https://doi.org/10.1101/2025.04.05.647371; this version posted May 31, 2025. The copyright holder for this preprint (which was not certified by peer review) is the author/funder, who has granted bioRxiv a license to display the preprint in perpetuity. It is made available under aCC-BY 4.0 International license.

We use only components that fall above the noise limit for the calculation of entropy. Again, we can use either their individual limits of detection, as in the main text, or an overall limit of detection. In Fig. S4b, we use the overall detection limit, and in Fig. S4c, we use the individual detection limit. Using entropy instead of a discrete number does not change the result in the main text, which is that the pleiotropic expansion hypothesis is not supported.

4. HOW DOES DIMENSIONALITY DEPEND ON NUMBER OF PERTURBATIONS?

The inferred dimensionality of a matrix via a low-rank approximation clearly depends on the rank of the input matrix. In particular, we have roughly 20 perturbations for each base environment, so we are doing a low-rank approximation of a matrix with at most rank 20. This means that we cannot infer a dimensionality that is higher than the rank. In other words,

$$\text{inferred } D \leq \min(\text{num. environments, num. mutants})$$

Here, the number of environments is always lower than the number of mutants. To understand the dependence of our dimensionality results in section I.D on the number of environments we included in the study, we randomly selected n environments from each base and performed the same procedure described in section I.D. In short, we took 100 random choices of n environments for each base, where $n = 1, ..., 16$, and did SVD on the sub-matrix for each base. Then we used the inferred noise to generate noise-only matrices, and did SVD on these noise-only matrices. Finally, we asked what fraction the variance in the data could be explained by the top noise component. This sets our limit of detection, and allows us to infer dimensionality. For each instance, we obtain a dimensionality as a function of n. In Fig. S5, we plot this inferred dimensionality as a function of n, where each point is colored by the base environment.

Often, the dimensionality saturates as we add more environments, supporting the hypothesis that the underlying spaces are indeed low-dimensional. Additionally, across a range of different mutant sets and evolution environments, for any given n, we still do not find that the evolution environment is consistently lower dimensional than the other two base environments.

5. SYNTHETIC DATA FOR DIFFERENT FITNOTYPE OVERLAP SCENARIOS

To verify our intuition and validate our analyses, we constructed a synthetic dataset assuming that hubs have different levels of overlap in their fitnotypes. We assume that mutations have random effects on a set of K latent fitnotypes, drawn from a standard Normal distribution. Each of these K fitnotypes will have a random, non-negative weight in an environment, drawn from a uniform distribution between $[0, 1)$. A base environment is characterized by which fitnotypes are relevant, and environmental perturbations will simply perturb the weights of these fitnotypes, but won't change their identity. In Fig. S6a, we show three example environment-to-fitnotype matrices, corresponding to the different scenarios of fitnotype overlap referenced in Fig. 5. In the first scenario, fitnotypes are equally important in both base environments. In the third scenario (orthogonal fitnotypes), fitnotypes that matter in Base 1 have 0 weight in Base 2, and vice versa. In the partially overlapping fitnotypes scenario, some fitnotypes are shared and others are private to one base or the other. The resulting fitness matrices are shown in Fig. S6b, obtained by multiplying the mutant-to-fitnotype matrix and the environment-to-fitnotype matrix together, and adding normally distributed noise with mean 0 and standard deviation 0.05. The separation between Base 1 and Base 2 grows increasingly obvious as there is less overlap in underlying fitnotypes. In Fig. S6c, we do SVD on each base separately and quantify the variance explained by each component (as we did in Fig. 4a. We can see that the number of relevant fitnotypes in each base is recovered by the elbow technique (finding the kink in the plot), likely due to the true linearity of the underlying data, and the low levels of noise (as opposed to the real data). Finally, we used the same linear regression technique described in Fig. 5a to assess fitnotypic overlap. We can see that qualitatively, we recover what we expect: equivalent prediction for fully overlapping fitnotype spaces, less predictive power for partially overlapping fitnotypes, and little to no prediction for orthogonal fitnotypes.

bioRxiv preprint doi: https://doi.org/10.1101/2025.04.05.647371; this version posted May 31, 2025. The copyright holder for this preprint (which was not certified by peer review) is the author/funder, who has granted bioRxiv a license to display the preprint in perpetuity. It is made available under aCC-BY 4.0 International license.

6. FITNOTYPE OVERLAP FOR OTHER MUTANT SETS

In Fig. S7, we perform the same analysis as in Fig. 5 in the main text, but for different groups of mutants. We find different patterns of overlap for different mutants, emphasizing the dependence of our approach on the constituent genotypes. Intriguingly, there are multiple instances where a target base is more predictable than the perturbations within the training base for certain groups of mutants. A further investigation of the genotype dependence of these patterns of overlaps would be interesting, but is outside the scope of this work.

7. PREDICTIONS SEPARATED OUT BY PERTURBATION

The linear regression procedure presented in Fig. 5a makes a prediction for δX of all mutants in our dataset, in a particular perturbation. That perturbation is either a test perturbation held out from the training base, or a test perturbation from a different base. The overall "predictive power" of our model is aggregated over predictions for all perturbations. In Fig. S8, we show the fraction of variance explained by each component of our model for each test perturbation. We see that indeed there is much heterogeneity in which perturbations are predictable. This suggests that different perturbations have different projections onto the relevant fitnotypes, and therefore are not all probing the same spaces.

8. COMPARISONS WITH DIFFERENT MODELS

In Fig. S9a, we compare the prediction accuracy of three different models for δX in two example environments. If the pleiotropic shift model is correct, we should be able to use it to make better predictions for δX across environmental bases than a model that does not consider fitnotypic diversity, or a model that does not incorporate base-dependence of these fitnotypes. The model that ignores fitnotypes, but has full genotypic and environmental information, could easily be very powerful, because it has information about how genes perform in the test environment. For each mutant included in Fig. 4, we know what the putative adaptive mutation was, and in which gene it landed. In many cases, we also have multiple mutations in the same gene, allowing use to calculate a mean δX effect across them. In Fig. S9a, the first column shows the predicted δX using the mean across the gene class (see color of point for gene information), plotted against the measured δX. The gene model is discrete, and assumes that all mutations in the same gene have the same fitness effect. While the model has a non-zero coefficient of determination, it is not highly predictive, and fails to resolve fitness differences between mutations that land in the same gene. The second column of Fig. S9a uses the δX effect for the perturbation in question, averaged across the other two bases. We saw in Fig. 3 that using δX from one base to predict δX in another base was not very accurate, but perhaps smoothing out the heterogeneity from different bases could make this a better predictor? This model is less discrete and can resolve different δX effects from mutations in the same gene, but it is even less predictive than the gene model overall.

Both models, however, do much worse at predicting δX in these two environments than a full linear model that allows for phenotypic diversity, and allows for re-weighting these phenotypes. In the final column, we use a model very similar to the one described in Eq. 5, but we actually predict the δX effect of held-out mutants using a bi-cross-validation approach (18, 36). Operationally, this means that the model is trained on neither the exact environment nor the exact mutants for whom we are attempting to predict δX. When we compare these models across all environments (Fig. 5d, we find that there is heterogeneity in the coefficient of determination, but on average our bi-cross-validation model does best at predicting δX. It is both more able to resolve subtle differences in fitness effect from mutations in the same gene, and it is able to flexibly re-weight the importance of fitnotypes across environments.

bioRxiv preprint doi: https://doi.org/10.1101/2025.04.05.647371; this version posted May 31, 2025. The copyright holder for this preprint (which was not certified by peer review) is the author/funder, who has granted bioRxiv a license to display the preprint in perpetuity. It is made available under aCC-BY 4.0 International license.

Environment Name	Base media	0.5M NaCl?	Glucose concentratic	Extra component?	Flask shape	Transfer time	Temperature	Shaking	Batch
2Day base	M3	No	1.50%	No	Normal	48 hours	30C	Yes	2,3,4
2Day_1.4	M3	No	1.40%	No	Normal	48 hours	30C	Yes	2
2Day_1.4Baffle	M3	No	1.40%	No	Baffle	48 hours	30C	Yes	2
2Day_1.8	M3	No	1.80%	No	Normal	48 hours	30C	Yes	2
2Day_1.8Baffle	M3	No	1.80%	No	Baffle	48 hours	30C	Yes	2
2Day_Suc	M3	No	1.50%	1% Sucrose	Normal	48 hours	30C	Yes	3
2Day_SucBaffle	M3	No	1.50%	1% Sucrose	Baffle	48 hours	30C	Yes	3
2Day_Raffinose	M3	No	1.50%	0.5% Raffinose	Normal	48 hours	30C	Yes	3
2Day_RaffinoseBaffle	M3	No	1.50%	0.5% Raffinose	Baffle	48 hours	30C	Yes	3
2Day_No Shake	M3	No	1.50%	No	Normal	48 hours	30C	No	3
2Day_0.5% EtOH	M3	No	1.50%	0.5% Ethanol	Normal	48 hours	30C	Yes	4
2Day_4uMH89	M3	No	1.50%	4 uM H89	Normal	48 hours	30C	Yes	4
2Day_10uMH89	M3	No	1.50%	10 uM H89	Normal	48 hours	30C	Yes	4
2Day_10uMParomomyci	M3	No	1.50%	10 uM Paromomycin	Normal	48 hours	30C	Yes	4
2Day_50uMParomomyci	M3	No	1.50%	50 uM Paromomycin	Normal	48 hours	30C	Yes	4
1Day Base	M3	No	1.50%	No	Normal	24 hours	30C	Yes	1,2,3,4
1Day 30Baffle	M3	No	1.50%	No	Baffle	24 hours	30C	Yes	1
1Day 32	M3	No	1.50%	No	Normal	24 hours	32C	Yes	1
1Day 32Baffle	M3	No	1.50%	No	Baffle	24 hours	32C	Yes	1
1Day 28	M3	No	1.50%	No	Normal	24 hours	28C	Yes	1
1Day_1.4	M3	No	1.40%	No	Normal	24 hours	30C	Yes	2
1Day_1.4Baffle	M3	No	1.40%	No	Baffle	24 hours	30C	Yes	2
1Day_1.8	M3	No	1.80%	No	Normal	24 hours	30C	Yes	2
1Day_1.8Baffle	M3	No	1.80%	No	Baffle	24 hours	30C	Yes	2
1Day_Suc	M3	No	1.50%	1% Sucrose	Normal	24 hours	30C	Yes	3
1Day_SucBaffle	M3	No	1.50%	1% Sucrose	Baffle	24 hours	30C	Yes	3
1Day_Raffinose	M3	No	1.50%	0.5% Raffinose	Normal	24 hours	30C	Yes	3
1Day_RaffinoseBaffle	M3	No	1.50%	0.5% Raffinose	Baffle	24 hours	30C	Yes	3
1Day_No Shake	M3	No	1.50%	No	Normal	24 hours	30C	No	3
1Day_0.5% EtOH	M3	No	1.50%	0.5% Ethanol	Normal	24 hours	30C	Yes	4
1Day_4uMH89	M3	No	1.50%	4 uM H89	Normal	24 hours	30C	Yes	4
1Day_10uMH89	M3	No	1.50%	10 uM H89	Normal	24 hours	30C	Yes	4
1Day_10uMParomomyci	M3	No	1.50%	10 uM Paromomycin	Normal	24 hours	30C	Yes	4
1Day_50uMParomomyci	M3	No	1.50%	50 uM Paromomycin	Normal	24 hours	30C	Yes	4
Salt Base	M3	Yes	1.50%	No	Normal	24 hours	30C	Yes	1,2,3
Salt 30Baffle	M3	Yes	1.50%	No	Baffle	24 hours	30C	Yes	1
Salt 32	M3	Yes	1.50%	No	Normal	24 hours	32C	Yes	1
Salt 32Baffle	M3	Yes	1.50%	No	Baffle	24 hours	32C	Yes	1
Salt 28	M3	Yes	1.50%	No	Normal	24 hours	28C	Yes	1
Salt_1.4	M3	Yes	1.40%	No	Normal	24 hours	30C	Yes	2
Salt_1.4Baffle	M3	Yes	1.40%	No	Baffle	24 hours	30C	Yes	2
Salt_1.8	M3	Yes	1.80%	No	Normal	24 hours	30C	Yes	2
Salt_1.8Baffle	M3	Yes	1.80%	No	Baffle	24 hours	30C	Yes	2
Salt_Suc	M3	Yes	1.50%	1% Sucrose	Normal	24 hours	30C	Yes	3
Salt_SucBaffle	M3	Yes	1.50%	1% Sucrose	Baffle	24 hours	30C	Yes	3
Salt_Raffinose	M3	Yes	1.50%	0.5% Raffinose	Normal	24 hours	30C	Yes	3
Salt_RaffinoseBaffle	M3	Yes	1.50%	0.5% Raffinose	Baffle	24 hours	30C	Yes	3
Salt_No Shake	M3	Yes	1.50%	No	Normal	24 hours	30C	No	3
Salt_0.5% EtOH	M3	Yes	1.50%	0.5% Ethanol	Normal	24 hours	30C	Yes	4
Salt_4uMH89	M3	Yes	1.50%	4 uM H89	Normal	24 hours	30C	Yes	4
Salt_10uMH89	M3	Yes	1.50%	10 uM H89	Normal	24 hours	30C	Yes	4
Salt_10uMParomomycin	M3	Yes	1.50%	10 uM Paromomycin	Normal	24 hours	30C	Yes	4
Salt_50uMParomomycin	M3	Yes	1.50%	50 uM Paromomycin	Normal	24 hours	30C	Yes	4

FIG. S1 **Table of environmental perturbation components** Here, we present a detailed description of each environment and perturbation. We color the rows by base environment, and specify all variants of the environment, along with which batch of the experiment included each perturbation.

bioRxiv preprint doi: https://doi.org/10.1101/2025.04.05.647371; this version posted May 31, 2025. The copyright holder for this preprint (which was not certified by peer review) is the author/funder, who has granted bioRxiv a license to display the preprint in perpetuity. It is made available under aCC-BY 4.0 International license.

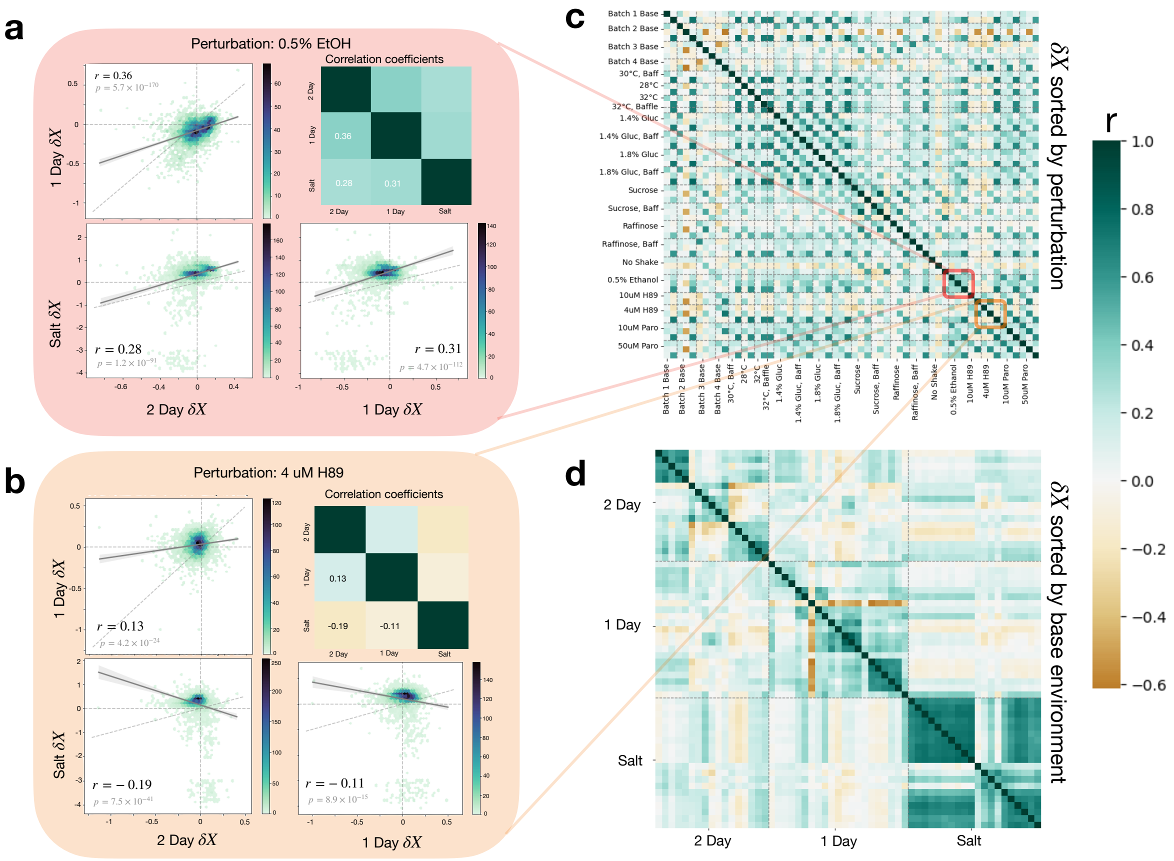

FIG. S2 **ExE interactions across all mutants**(a) 2 dimensional histograms of δX due to focal perturbation on two different base environments. Color of pixel corresponds to number of mutants in the bin. Top right shows correlation coefficient for each environmental comparison (b) Correlation matrix between environmental perturbations, clustered by perturbation (then batch) (top) and base environment (bottom). Block diagonal form is more apparent on the bottom, suggesting that the base environment is important for determining δX.

bioRxiv preprint doi: https://doi.org/10.1101/2025.04.05.647371; this version posted May 31, 2025. The copyright holder for this preprint (which was not certified by peer review) is the author/funder, who has granted bioRxiv a license to display the preprint in perpetuity. It is made available under aCC-BY 4.0 International license.

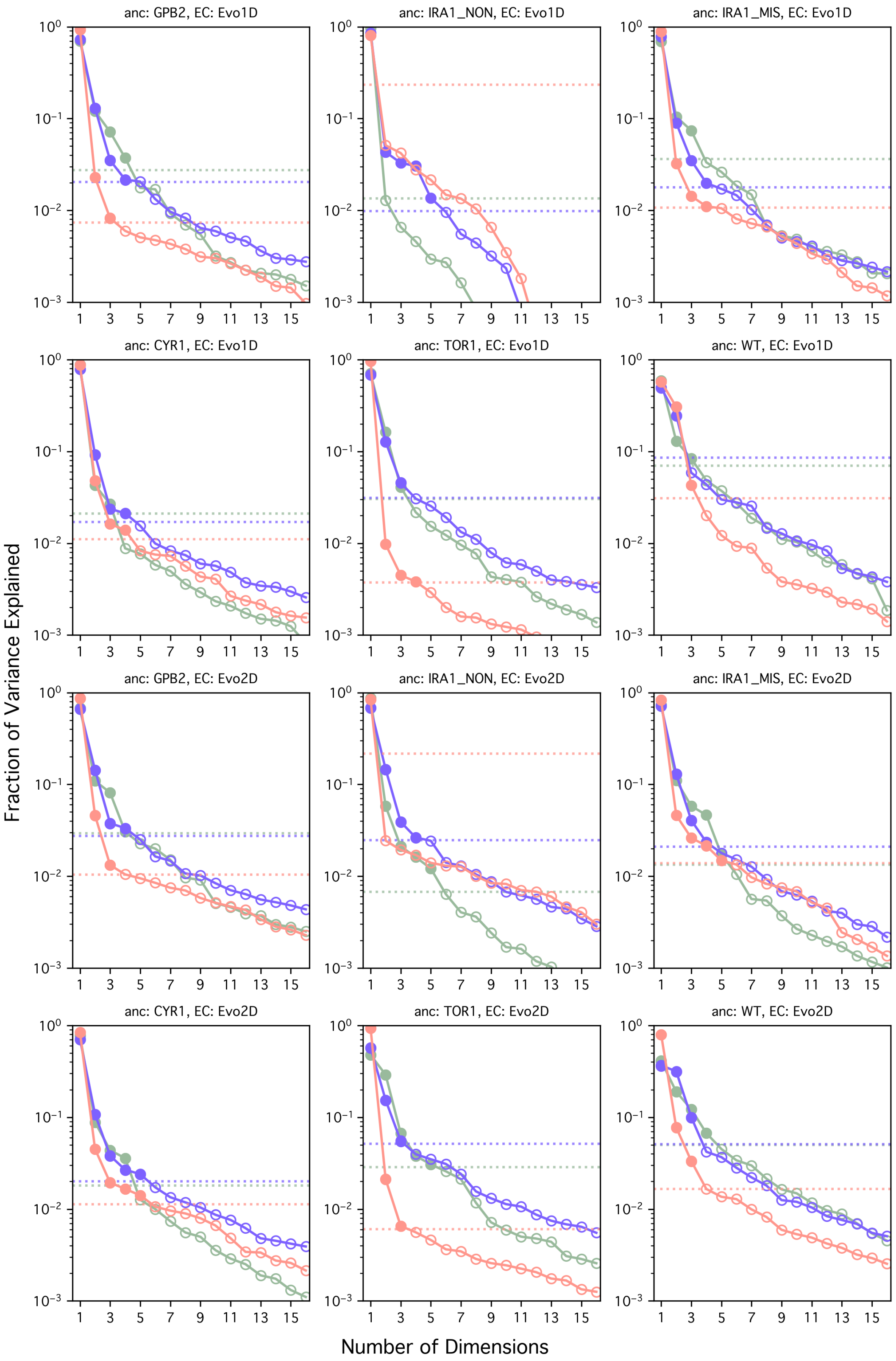

FIG. S3 **Scree plots for second step mutants** Each set of mutants has a different ancestor, and a different evolution condition. We did SVD on each set of mutants in each base environment, and here show the fraction of variance explained by each component for each base. Green is 2 Day, blue is 1 Day, and pink is Salt.

bioRxiv preprint doi: https://doi.org/10.1101/2025.04.05.647371; this version posted May 31, 2025. The copyright holder for this preprint (which was not certified by peer review) is the author/funder, who has granted bioRxiv a license to display the preprint in perpetuity. It is made available under aCC-BY 4.0 International license.

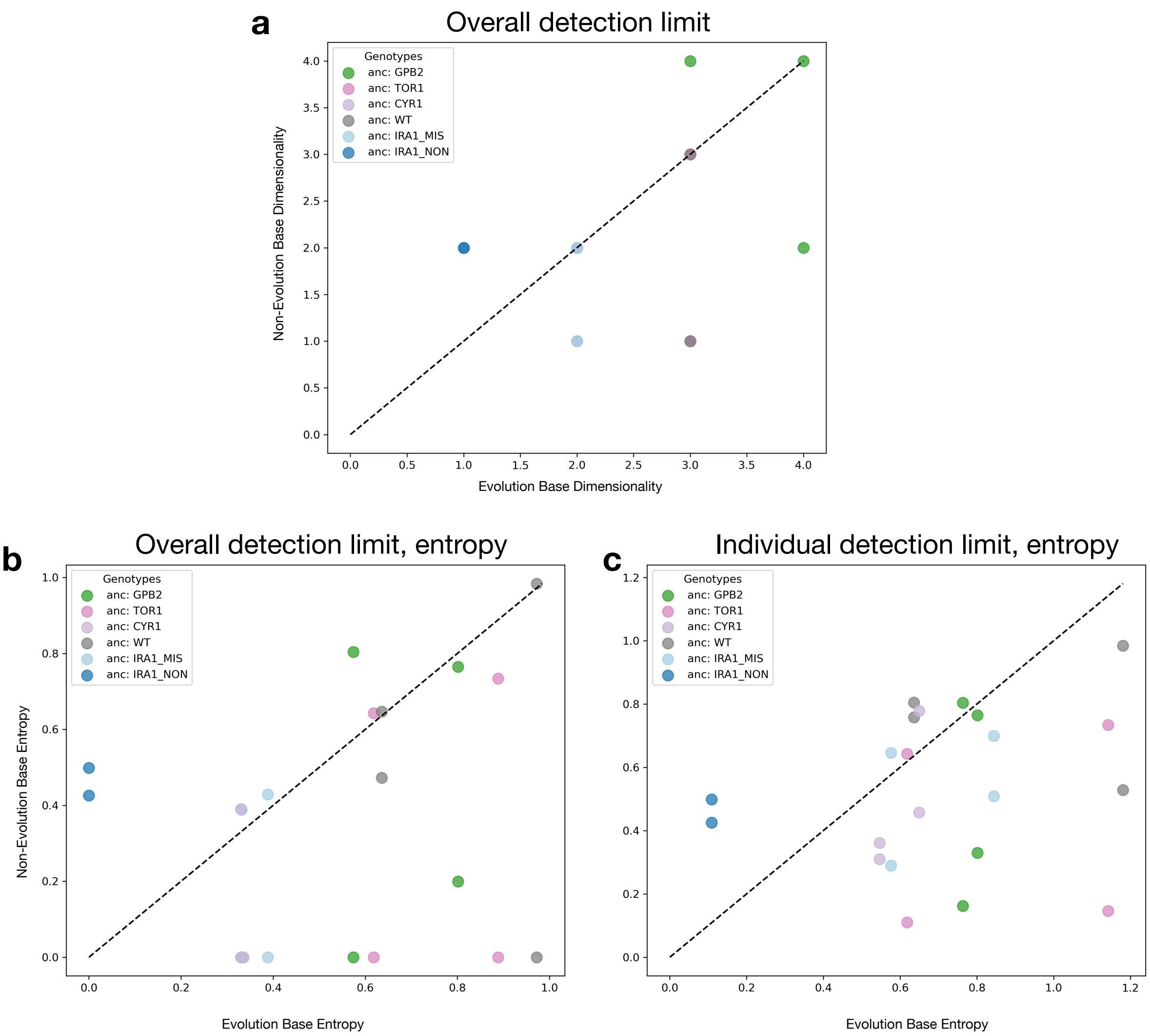

FIG. S4 **Quantifying dimensionality in alternate ways** Inferred dimensionality in evolution environment base plotted versus dimensionality inferred in alternate base. (a) Dimensionality is inferred based on how many components fall above the most explanatory noise-only matrix for all the bases. (b) Entropy of the distribution of variance explained for the components that fall above the overall limit of detection across bases is used as a proxy for dimensionality. (c) Entropy of distribution of variance explained for components that explain more than each base's individual detection limit is used as a proxy for dimensionality.

bioRxiv preprint doi: https://doi.org/10.1101/2025.04.05.647371; this version posted May 31, 2025. The copyright holder for this preprint (which was not certified by peer review) is the author/funder, who has granted bioRxiv a license to display the preprint in perpetuity. It is made available under aCC-BY 4.0 International license.

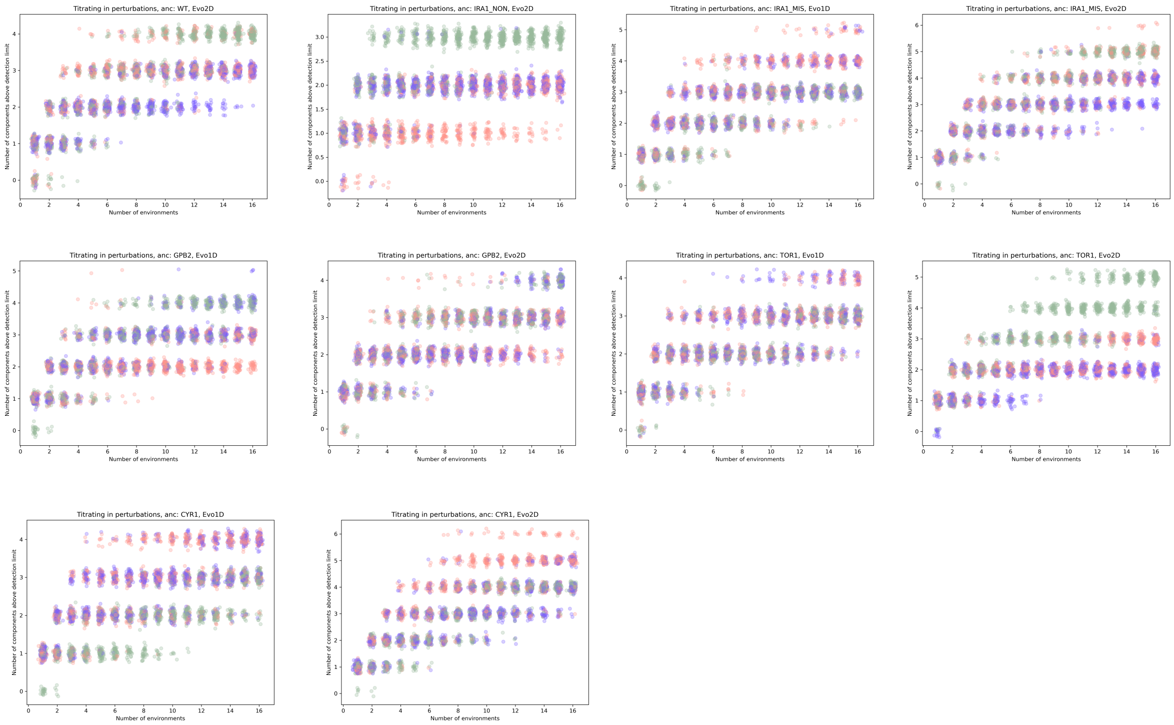

FIG. S5 **Titrating in perturbations and inferring dimensionality** Each panel is a distinct set of mutants, evolved from a different ancestor and in a different environment. Across a range of n, we still find that the evolution condition is not systematically lower in inferred dimensionality than the other two base environments. X and Y jitter added.

bioRxiv preprint doi: https://doi.org/10.1101/2025.04.05.647371; this version posted May 31, 2025. The copyright holder for this preprint (which was not certified by peer review) is the author/funder, who has granted bioRxiv a license to display the preprint in perpetuity. It is made available under aCC-BY 4.0 International license.

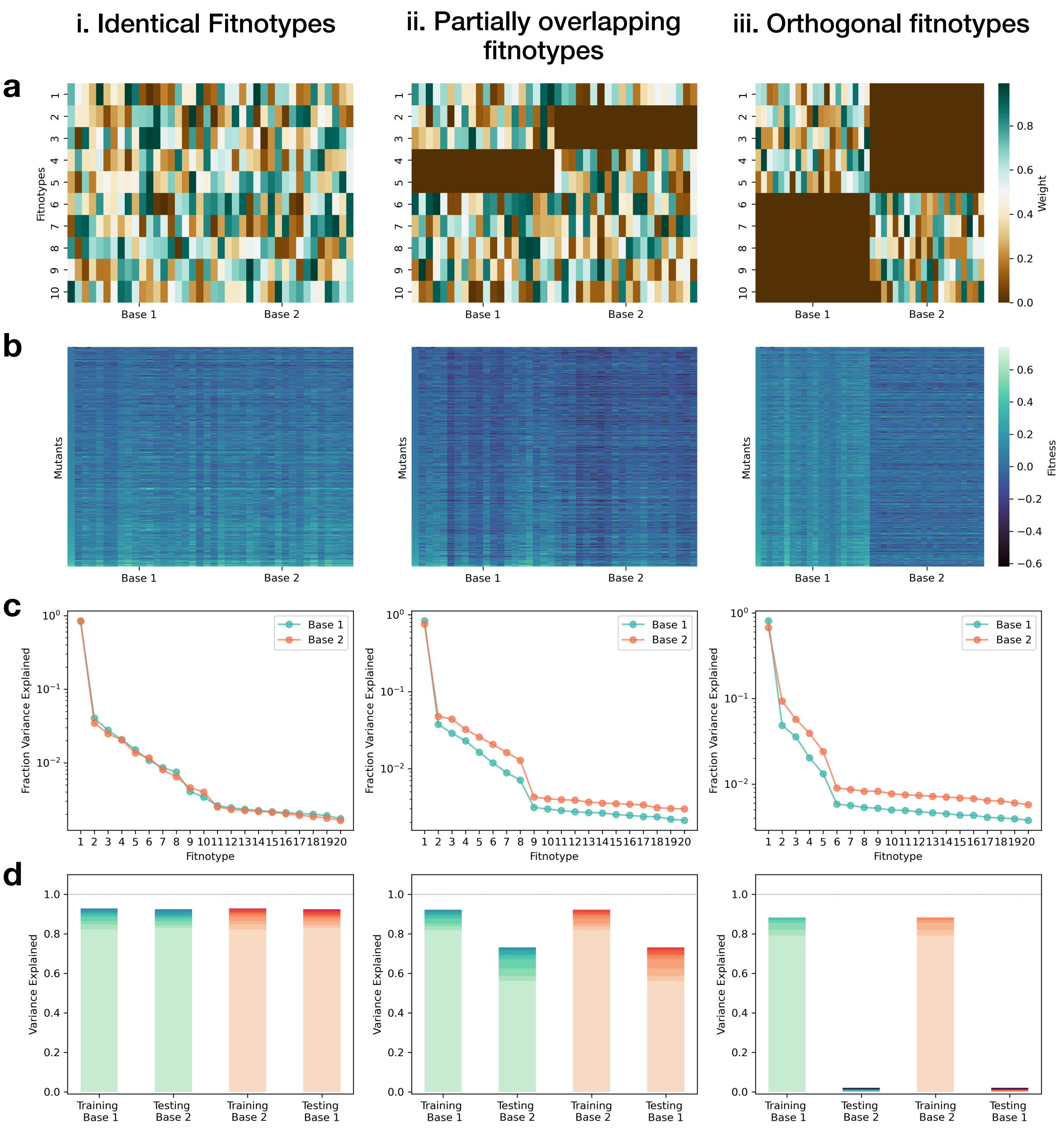

FIG. S6 **Synthetic data** (a) Underlying weights of fitnotypes (rows) in different environments (columns) for different scenarios of fitnotype overlap. (b) Fitness matrices for the same mutant-to-fitntotype matrix, but different environment-to-fitnotype matrices (from panel a). (c) Variance explained by each inferred fitnotype using SVD to identify fitnotypes. (d) Prediction within and across bases for different fitntoype overlap.

bioRxiv preprint doi: https://doi.org/10.1101/2025.04.05.647371; this version posted May 31, 2025. The copyright holder for this preprint (which was not certified by peer review) is the author/funder, who has granted bioRxiv a license to display the preprint in perpetuity. It is made available under aCC-BY 4.0 International license.

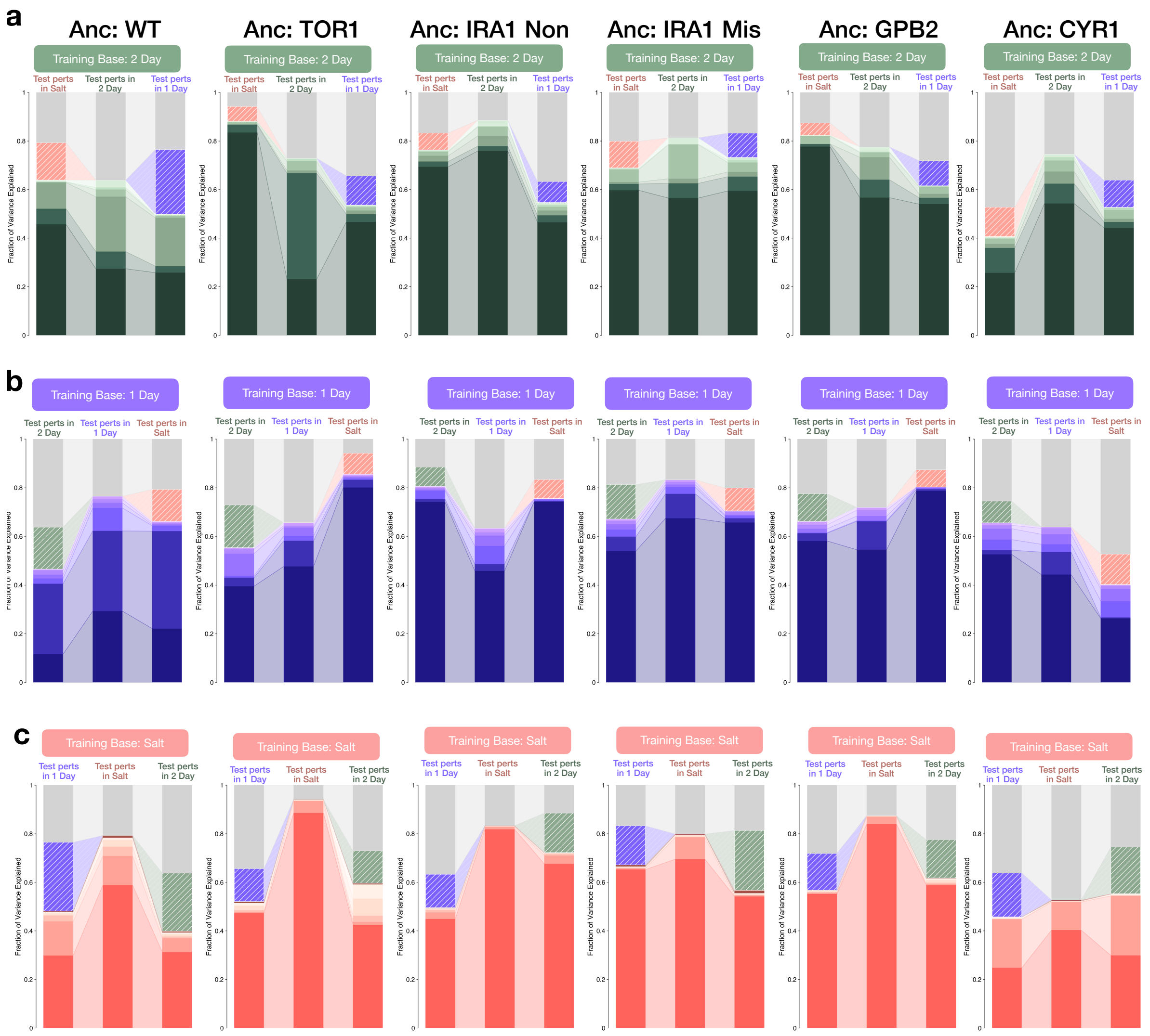

FIG. S7 **Predicting δX with linear regression for different mutant sets** Columns correspond to predictions for different mutant sets (mutants that evolved from different ancestors). We show results for training base 2 Day (a), training base 1 Day (b), and training base Salt (c).

bioRxiv preprint doi: https://doi.org/10.1101/2025.04.05.647371; this version posted May 31, 2025. The copyright holder for this preprint (which was not certified by peer review) is the author/funder, who has granted bioRxiv a license to display the preprint in perpetuity. It is made available under aCC-BY 4.0 International license.

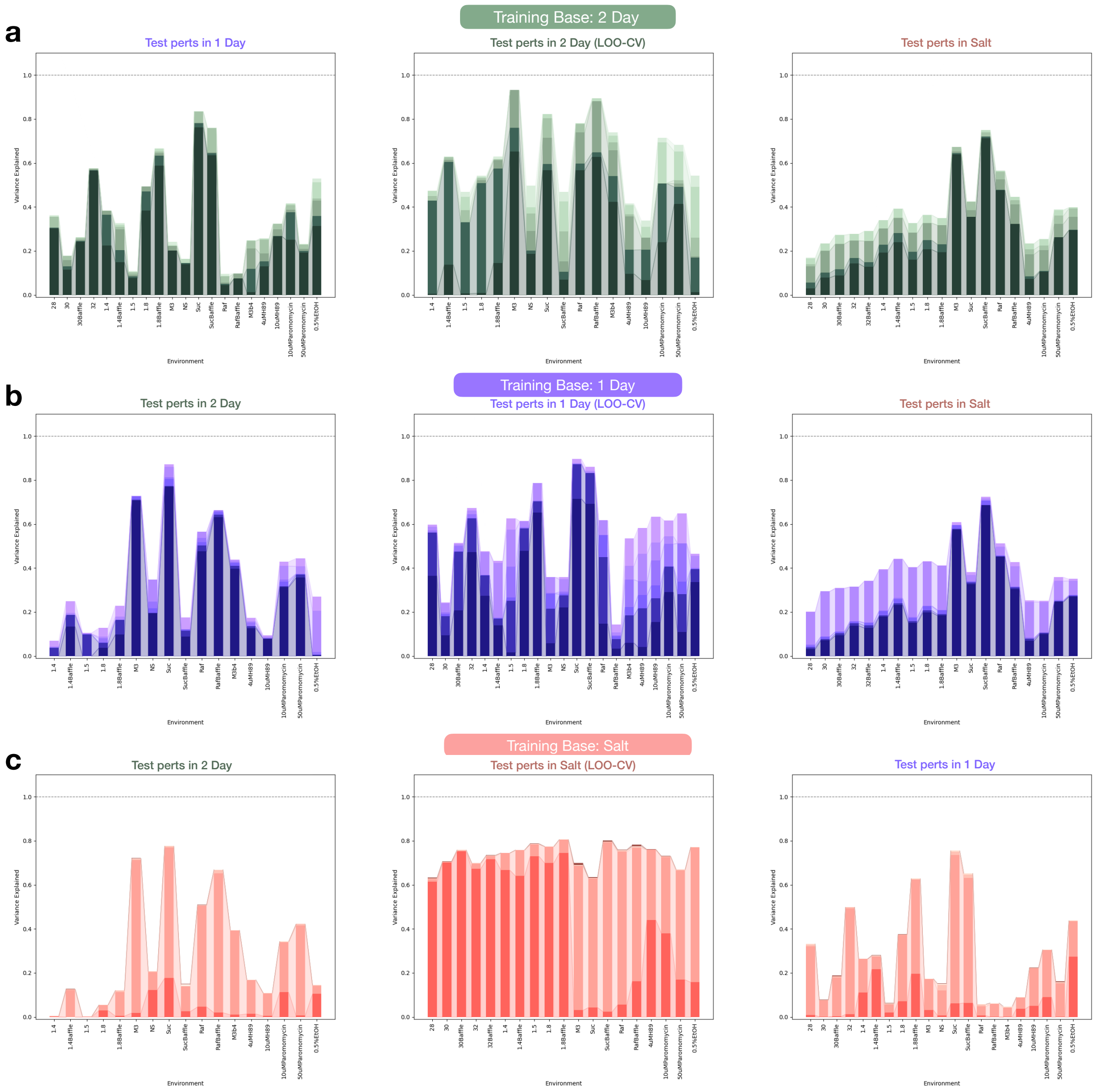

FIG. S8 **Predictions for target bases separated out by perturbation** Each prediction is done for one perturbation. The contribution of each component from the training base to predicting δX in the test perturbation is shown here. (a) Training base is 2 Day. (b) Training base is 1 Day. (c) Training base is Salt.

bioRxiv preprint doi: https://doi.org/10.1101/2025.04.05.647371; this version posted May 31, 2025. The copyright holder for this preprint (which was not certified by peer review) is the author/funder, who has granted bioRxiv a license to display the preprint in perpetuity. It is made available under aCC-BY 4.0 International license.

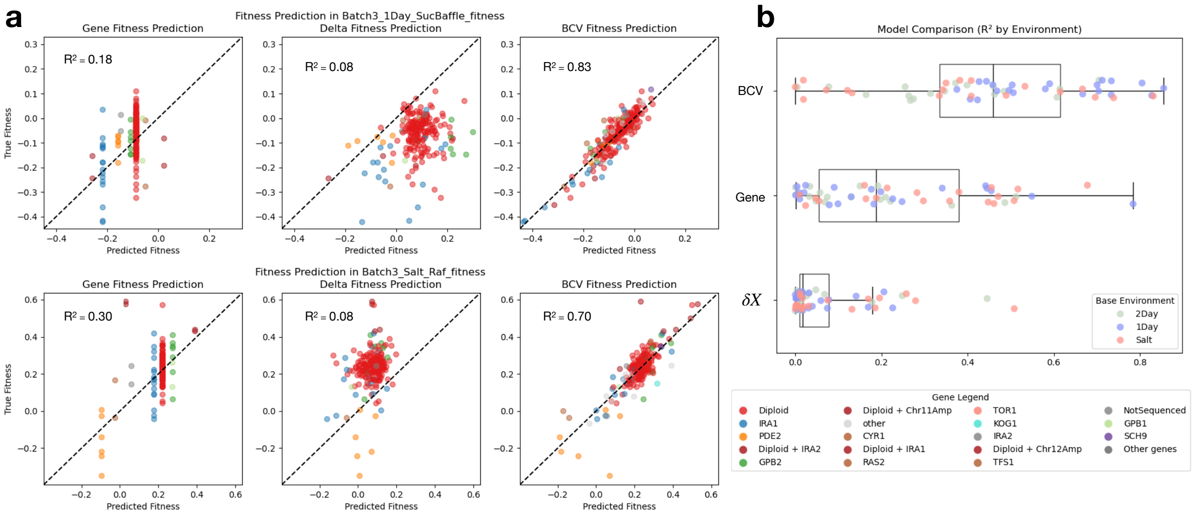

FIG. S9 (a) Comparison of model predictions and measured δX for 2 Day adaptive mutants, using a gene-only model, a perturbation-only model, and a linear fitnotype model (BCV), for two environments. (b) R^2 for all environments for each model, colored by base environment. The δX is least predictive on average, and the BCV is most predictive on average, despite heterogeneity.
